# Role of PARP Inhibitors: A New Hope for Breast Cancer Therapy

**DOI:** 10.3390/ijms26062773

**Published:** 2025-03-19

**Authors:** Kamalendu De, Malabendu Jana, Bhabadeb Chowdhury, Gloria M. Calaf, Debasish Roy

**Affiliations:** 1 Department of Biological Sciences (Botany), Midnapore City College, Midnapore 721129, West Bengal, India; kamalendude530@gmail.com; 2 Department of Neurological Science, Rush University School of Medicine, Chicago, IL 773, USA; malabendu_jana@rush.edu; 3 HIV Dynamics and Replication Program, National Institute of Health, Frederick, MD 21702, USA; bhabadeb.chowdhury@nih.gov; 4 Instituto de Alta Investigación, Universidad de Tarapacá, Arica 1000000, Chile; 5 Department of Natural Sciences, Hostos College of The City University of New York, Bronx, NY 718, USA; droy@hostos.cuny.edu

**Keywords:** tumors, breast cancer, *BRCA1*, *BRCA2*, PARP inhibitors, therapy

## Abstract

Tumors formed by the unchecked growth of breast cells are known as breast cancer. The second most frequent cancer in the world is breast cancer. It is the most common cancer among females. In 2022, 2,296,840 women were diagnosed with breast cancer. The therapy of breast cancer is evolving through the development of Poly (ADP-ribose) polymerase (PARP) inhibitors, which are offering people with specific genetic profiles new hope as research into the disease continues. It focuses on patients with *BRCA1* and *BRCA2* mutations. This review summarizes the most recent research on the mechanisms of action of PARP inhibitors and their implications for breast cancer therapy. We review how therapeutic applications are developing and highlight recent studies showing the effectiveness of these medicines whether used alone or in combination. Furthermore, the significance of customized therapy is highlighted in enhancing patient outcomes as we address the function of genetic testing in identifying candidates for PARP inhibition. Recommendations for future research areas to maximize the therapeutic potential of PARP inhibitors are also included, along with challenges and limits in their clinical usage. The objective of this review is to improve our comprehension of the complex interaction between breast cancer biology and PARP inhibition. This knowledge will help to guide screening approaches, improve clinical practice, and support preventive initiatives for people at risk.

## 1. Introduction

Breast cancer is a major worldwide health concern. Globally, breast cancer is the most often diagnosed cancer. The four types of breast cancer are identified by distinct histological and pathological characteristics: luminal A, luminal B, HER2 overexpression, and triple-negative breast cancer (TNBC) [1]. In 2022, there were 2,296,840 new cases reported, and it was the fourth leading cause of mortality, with 666.103 deaths [2]. The majority of women with breast cancer are diagnosed between the ages of 50 and 69 [3]. Higher risks are associated with factors including alcohol intake, obesity, and physical inactivity [4].

Breast cancer rates are greater in industrialized nations including the United States, Canada, and some Western European countries [5]. Numerous variables, including lifestyle and screening habits, are sometimes blamed for this [6]. Urbanization and changes in lifestyle have contributed to a notable increase in breast cancer in emerging nations [7]. It has been demonstrated that routine screening lowers death rates by facilitating early breast cancer identification [8]. There are significant differences in access to screening; industrialized countries usually have greater screening rates and better access than underdeveloped countries [9]. Better survival rates result from major therapy advancements, such as immunotherapies and targeted medicines. In high-income nations, the 5-year survival rate for breast cancer is around 90%; however, in low- and middle-income nations, this number may be lower owing to late-stage detection and fewer treatment choices [10]. Global campaigns are raising awareness of breast cancer and providing financing for research, which will improve detection methods and treatment choices. Mutations in the PI3K/Akt/mTOR pathway, which is also crucial in cell proliferation and survival, can accelerate the development of breast cancer [11].

Erb-b2 receptor tyrosine kinase 2 (ERBB2), progesterone receptor (PGR), and estrogen receptor (ER) are three key receptors frequently used to classify breast cancers. TNBC is a subtype of breast cancer characterized by the absence of these receptors [12]. Cancer cells grow in response to estrogens when the ER is positive. Nevertheless, TNBC lacks this receptor. PGRs, which are linked to the hormone progesterone, are likewise absent from TNBC. When compared to other breast cancer subtypes, TNBC is more aggressive and is linked to a worse prognosis and increased chance of recurrence [13]. Younger women and women of African heritage are more likely to experience it [14]. Treatment options for TNBC are more restricted since the disease does not respond to hormone treatments or medications that target HER2 [15]. Chemotherapy is usually part of standard treatment, and researchers are also investigating targeted treatments and immunotherapies. The molecular features of TNBC are being investigated by researchers to find possible targeted medicines and biomarkers that may predict the course of therapy. Novel approaches to immunotherapy and clinical trials hold great promise for improving the prognosis of TNBC patients [16].

A new taxonomy of breast cancer based on genetic characteristics was proposed by several studies using microarray analysis. At least five molecular breast cancer subtypes have been identified as a result of gene expression microarray-based class discovery studies: basal-like, HER2, normal breast-like, luminal A, and luminal B [17,18,19,20,21,22]. Microarray research reveals the strongest difference between the transcriptomes of ER-negative (ER) and estrogen receptor-positive (ERþ) breast cancers. Among the molecular subtypes of breast cancer determined by gene expression profiling studies, the basal-like group is the focus of controversy. The differences between triple-negative and basal-like breast cancers are important to pathologists, and the characteristics of basal-like breast cancer have been studied [21,23,24,25]. There are few direct comparisons between the molecular subtypes described by microarrays and immunohistochemistry markers [26,27]. There is no widely recognized definition for basal-like breast cancers. Various groups have attempted to define basal-like breast cancers using panels of immunohistochemical markers and microarray-based expression profiling [28], including (1) the triple-negative immunophenotype (absence of ER, PGR, and HER2 expression); (2) the expression of one or more high-molecular-weight/basal cytokeratins (CK5/6, CK14, and CK17); (3) the absence of ER and HER2 expression combined with CK5/6 and/or EGFR expression [26], and (4) the absence of ER, PGR, and HER2 expression in combination with CK5/6 and/or EGFR expression. These cancers exhibit unique clinical manifestations [29], including histological features [27,30] and chemotherapeutic response [18,20,31,32,33,34,35,36,37].

Approximately 15% of all breast cancers are basal-like tumors, which primarily affect African-American women and young patients [14]. Most basal-like breast cancers lack or show low levels of ER and PGR, and lack HER2 protein overexpression and HER2 gene amplification, while expressing genes and proteins typically found in “basal” or myoepithelial cells of the normal breast, such as high-molecular-weight cytokeratins (5/6, 14 and 17) [26,28,30,38], P-cadherin [39], caveolins [40,41], and EGFR [26], and, in a minority of cases, harboring EGFR gene amplification [42] or aneusomy [43].

Up to 85% of patients have TP53 gene mutations or p53 immunohistochemistry expression [44,45], and these malignancies commonly involve changes to the pRB and p16 G1/S cell cycle checkpoint [46,47]. In contrast to tumors of other molecular subtypes, a study showed that about 30% of basal-like breast cancers concurrently exhibit overexpression of p16 and p53 immunoreactivity (pRB/p16þ/p53þ) and a lack of pRB expression [46]. Contrary to “basal” or myoepithelial cells of a normal breast, basal-like breast cancers almost uniformly express cytokeratins 8 and/or 18, raising doubts about the initial histogenetic implications of the microarray-based taxonomy of breast cancers from basal/myoepithelial cells [23,48]. Basal-like cancers also exhibit remarkably high proliferation indices, as determined by mitotic counting or by the Ki67 labeling index [33,46,49].

Nonetheless, there is broad consensus that triple-negative cancers are tumors that do not express ER, PGR, or HER2. Depending on the criteria used to define ER and PGR positivity and the techniques employed for HER2 assessment, these tumors make up 10–17% of all breast carcinomas [32,34,50,51,52,53,54,55,56,57]. The ASCO/CAP guidelines have changed the classification of HER2 and hormone receptor positivity, which may somewhat alter prevalence rates for triple-negative breast tumors in future research [58,59]. When compared to patients with non-basal-like/non-triple-negative controls, patients with triple-negative tumors [51,55], like those with basal-like cancers [38], have a noticeably lower survival rate after the initial metastatic event.

Although there are many parallels between triple-negative and basal-like breast cancers, the labels are not interchangeable, despite people using them interchangeably in the past [21,24,44,60,61]. Most tumors expressing “basal” markers are triple-negative [25,26,28,44,52,62], and most triple-negative malignancies do have a basal-like phenotype [31,52,60]. However, not all triple-negative tumors exhibit a basal-like phenotype by expression array analysis, and not all basal-like cancers, identified by gene expression profiling, lack ER, PGR, and HER2 [21,24,25,60,62,63,64,65,66].

By using gene expression profiling, the authors of [62] demonstrated that only 71% of triple-negative malignancies were of the basal-like subtype and that only 77% of molecular basal-like tumors were triple-negative. Similar findings were noted by de Ronde et al. and Parker et al. [22,66], who found that 18–40% of basal-like breast tumors identified by gene expression profiling did not exhibit a triple-negative phenotype, while 8–29% of triple-negative cancers did not exhibit a basal-like subtype by expression array analysis. The triple-negative phenotype is not a good surrogate marker for basal-like breast tumors [19,52,62].

Regarding the connection between the BRCA1 DNA repair associated (*BRCA1*) germ-line mutations and basal-like breast cancer, there is mounting evidence that basal-like breast tumors and the *BRCA1* pathway are related [67,68]. Most tumors of *BRCA1* germ-line mutation carriers, especially those identified before the age of 50, share morphological characteristics with basal-like cancers [69,70] and exhibit a basal-like phenotype as determined by immunohistochemistry [71,72] or expression arrays [19]. When compared to sporadic breast carcinomas and the BRCA2 DNA repair associated (*BRCA2*) mutant tumors, basal-like breast cancers and tumors arising in carriers of the *BRCA1* germ-line mutation exhibit a peculiar pattern of cell cycle protein expression [45,69,70,73]. They both rarely harbor *CCND1* gene amplification [45,70], but they express lower levels of p27 and higher levels of Skp2, cyclin E, and caspase-3 [69,73]. The molecular genetic profiles of sporadic basal-like malignancies are comparable to those of tumors that arise in carriers of *BRCA1* mutations, although they do not have *BRCA1* somatic mutations [74,75,76,77,78]. This is partly because these tumors have a malfunctioning *BRCA1* pathway [67,68,79].

In breast cancer, the Wnt/β-catenin signaling pathway is important [80]. Numerous biological functions, such as cell division, proliferation, and survival, depend on this route. A critical component of the signaling cascade of Wnt signaling, which plays a major role in cellular processes, is the β-catenin pathway. The cytoplasmic accumulation and stabilization of β-catenin occur as a result of Wnt proteins binding to their receptors on cell surfaces. Upon translocating to the nucleus, this β-catenin engages in transcription factor-mediated gene expression regulation affecting cellular processes like survival, differentiation, and proliferation. The development and spread of breast cancer are linked to the dysregulation of Wnt/β-catenin signaling [81].

Uncontrolled cell survival and proliferation may result from abnormal activation of this mechanism. The process of epithelial–mesenchymal transition (EMT), which is facilitated by metastasis, is mediated by Wnt/β-catenin signaling [81]. In TNBC, the Wnt/β-catenin signaling may be especially important, as its activation is frequently linked to aggressive tumor activity. Owing to its involvement in cancer, the Wnt/β-catenin pathway is investigated as a possible target for therapy. To stop tumor development and metastasis, researchers are looking at ways to block this route or its consequences. Because of its intricacy, and because Wnt signaling is essential for regular physiological activities, targeting the Wnt/β-catenin pathway is difficult. Overall, the Wnt/β-catenin signaling pathway affects the growth and progression of tumors, making it a crucial component of the biology of breast cancer [82].

The *BRCA1* and *BRCA2* genes, which stand for breast cancer gene 1 and gene 2, produce proteins that aid in the repair of damaged DNA in normal conditions [83]. Each gene is found in two copies in every person: one copy from each parent. Individuals who inherit a deleterious alteration (also known as a mutation or pathogenic variation) in one gene are more likely to develop several malignancies, including ovarian and breast cancers, among others [84]. Mutations in *BRCA1* and *BRCA2* are mainly inherited, so parents pass them on to their offspring. A genetic predisposition to breast cancer is linked to the *BRCA1* and *BRCA2* genes, with an ongoing danger of 40–60% in *BRCA1* mutation carriers and 13–30% in *BRCA2* alterations carriers [85,86].

There is a greater risk associated with *BRCA1* than *BRCA2* [87]. The majority of people with a detrimental mutation in either the *BRCA1* or *BRCA2* gene from one parent also have a healthy copy of the gene from the other parent. They will have one healthy copy of each gene to prevent cells from developing into cancer. However, during a person’s lifespan, the typical copy may alter or disappear. This modification refers to somatic alteration. When a somatic modification occurs in the single normal copy of one gene, the capacity of the cell to repair DNA is compromised, and it may develop into cancer [88]. Autosomal dominant inheritance is frequently observed in cases of *BRCA1* and *BRCA2* mutations [89]. This implies that the chance of acquiring malignancies, particularly breast and ovarian cancers, can be raised by receiving even one mutant copy of the gene from either parent. BRCA mutations may be indicated by a strong family history of pancreatic, prostate, ovarian, or breast malignancies [90].

There may be an inherited mutation in families where several relatives have been afflicted by these malignancies. Certain *BRCA1* and *BRCA2* mutations are more common in some groups, such as Ashkenazi Jews [91]. This is because of the founder effect, which is the genetic features of a small population amplified in their offspring. Although the majority of *BRCA* mutations are inherited, they can also develop on their own during the lifetime of a person because of environmental influences or mistakes in DNA replication; however, this is less common. Growing older can increase mutations because aging causes more cellular divisions, which raises the possibility of mistakes [92]. Knowing these variables can assist in estimating cancer risk and deciding if genetic testing is suitable for certain people or families. Most *BRCA1* and *BRCA2* mutations are inherited; that is, they are not brought on by alterations in lifestyle or exposure to toxins in the environment. Those with these genetic changes already have an increased risk of cancer due to environmental factors and lifestyle choices. Environmental contaminants and lifestyle variables (such as food, exercise, and smoking) might raise one’s overall chance of acquiring cancer [93].

These variables may further increase the risk of breast, ovarian, or other cancers in people with BRCA mutations. DNA damage can be caused by radiation, certain compounds, and environmental contaminants [94]. They may not directly cause *BRCA* mutations, but they can accelerate the growth of cancer in cells that already have genetic predispositions or mutations. In those with BRCA mutations, leading a healthy lifestyle may help lower their chance of developing cancer in general. This includes minimizing alcohol intake, abstaining from tobacco, eating a balanced diet, and exercising often. Although studies on the impact of environmental and lifestyle variables on cancer are ongoing, there is little proof to support that they might produce *BRCA1* or *BRCA2* mutations on their own. Poly (ADP-ribose) polymerase (PARP) inhibitors have emerged as a promising therapeutic strategy in the treatment of breast cancer, particularly for patients with hereditary *BRCA1* and *BRCA2* mutations [95]. These inhibitors exploit the concept of “synthetic lethality”, targeting cancer cells’ reliance on PARP for DNA repair [96].

*PARP1*/2 inhibitors are small-molecule compounds that block *PARP1* activity by competing with NAD^+^ for the active site. Interest in PARP inhibitors has increased since blocking *PARP1* results in notable cell death in cancer cells with gene mutations, including *BRCA1* and *BRCA2*. A genetic mutation that results in cell death and the combined effects of a drug cause synthetic lethality. By inhibiting PARP, these drugs hinder the ability of cancer cells to repair DNA damage, leading to increased cell death and offering a novel approach to managing this aggressive disease. PARP inhibitors are changing the face of breast cancer treatment as research advances, giving individuals with certain genetic profiles fresh hope [97]. Research is focused on understanding the mechanisms underlying PARP inhibitors and identifying effective treatment strategies that target this system. It seeks to deepen our understanding of the complex features of breast cancer and suggests further investigation, screening guidelines, and preventive measures.

## 2. The *BRCA1* Gene

The *BRCA1* gene, found on chromosome 17, is important for preserving genomic stability [98]. Through a process known as homologous recombination, it encodes a protein that aids in the repair of DNA breaks [99]. The prevention of the build-up of genetic mutations that can increase cancer is made possible by this process. Additionally, *BRCA1* is engaged in several cellular functions, such as apoptosis and cell cycle regulation [1]. In addition to regulating the cell cycle and apoptosis, *BRCA1* is essential for maintaining genomic stability and ensuring that DNA damage is properly repaired. *BRCA1* participates in the DNA damage response in cell cycle regulation, particularly at the G1/S and G2/M checkpoints [100]. Before allowing the cell to continue through the cell cycle, it aids in determining whether the DNA is intact. When damage is identified, *BRCA1* can assist in initiating signaling pathways that cause the cell cycle to stop, giving the body time to heal [101]. Cyclins and cyclin-dependent kinases (CDKs), which are essential for the progression of the cell cycle, interact with *BRCA1* [102].

By preventing cells from proliferating while their DNA is damaged, this interaction can help control how the cell cycle transitions between different phases. *BRCA1* guarantees that the cell only advances through the cycle once the DNA is intact by aiding in the repair of DNA double-strand breaks through homologous recombination [103]. When DNA damage is irreversible, *BRCA1* can induce apoptosis to get rid of potentially cancerous cells [104]. It aids in the activation of pathways that result in programmed cell death, stopping the spread of mutations. In response to severe DNA damage, *BRCA1* can enhance the ability of a cell to undergo apoptosis by regulating the expression and activity of several pro-apoptotic proteins [105].

*BRCA1* functions as a tumor suppressor, lowering the risk of tumorigenesis in tissues where it is normally expressed, by promoting apoptosis in damaged cells. The function of *BRCA1* in controlling the cell cycle and apoptosis highlights how important it is for preserving cellular integrity and halting the growth of cancer. The risk of developing ovarian and breast cancers is markedly elevated in individuals with mutations in the *BRCA1* gene. *BRCA1* mutation carriers are more likely than the general population to develop these cancers [106]. People with a family history of breast or ovarian cancer are often advised to undergo genetic testing for *BRCA1* mutations so preventive measures can be implemented and early detection occurs [107]. The wider effects of *BRCA1* in other cancers and its potential as a therapeutic target are still being investigated.

## 3. The *BRCA2* Gene

Similar to *BRCA1*, the *BRCA2* gene is found on chromosome 13 and is essential for preserving genomic stability [108]. It encodes a protein necessary for homologous recombination, a crucial step in precise DNA repair, to repair double-strand breaks in DNA. By supporting and directing the RAD51 protein (RAD51 homology1), which is necessary for the homologous recombination repair pathway, *BRCA2* aids in the repair of DNA damage [109]. By ensuring that cells can precisely repair damaged DNA, this process stops mutations that may eventually result in cancer. During the S and G2 phases, *BRCA2* plays a role in cell cycle checkpoint control, preventing cells from dividing until DNA damage is repaired [110].

The *BRCA2* gene is essential for preserving DNA integrity and preventing the development of cancer. The risk of developing breast, ovarian, and other cancers is greatly increased by mutations in *BRCA2* [106]. Like those with *BRCA1* mutations, people with *BRCA2* mutations are more likely to develop these cancers than the general population. Studies reveal a correlation between *BRCA2* mutations and various other cancer types, such as pancreatic and prostate cancers [111]. The detection of these mutations can direct prophylactic surgeries and enhanced surveillance treatment options such as targeted therapies that take advantage of the vulnerabilities of the cancer cells in DNA repair. This has important ramifications for genetic counseling and cancer treatment approaches. For those with a family history of breast or ovarian cancer, genetic testing for *BRCA2* mutations is frequently necessary.

## 4. The Interaction Between *BRCA1* and *BRCA2* Genes

In the biological mechanisms that preserve genomic stability, *BRCA1* and *BRCA2* collaborate closely, especially with DNA repair [112]. Effective homologous recombination, a critical mechanism for mending DNA double-strand breaks, depends on their interaction. The pathway of homologous recombination repair requires both *BRCA1* and *BRCA2*. While *BRCA2* is mainly in charge of the later stages of the DNA damage response, particularly stabilizing RAD51, a protein essential to the repair process, *BRCA1* is involved in the early stages of the response [1]. Signal transducer *BRCA1* is attracted to DNA damage sites and performs its function [101]. Once there, it can aid in the recruitment of RAD51 and *BRCA2* to the damaged site, facilitating the effective repair of DNA [109].

Both proteins are involved in the control of cell cycle checkpoints. While *BRCA2* ensures that the repair mechanisms are working properly before the cell divides, *BRCA1* can stop the cell cycle to make time for repair. With other elements involved in DNA repair, *BRCA1* and *BRCA2* can form protein complexes that coordinate their actions. To improve their cooperative function in repair pathways, they can interact with other repair proteins such as PALB2 (partner and localizer of BRACA2) and XRCC1 (X-ray repair cross-complementing Protein1), which serves as a bridge between *BRCA1* and *BRCA2* [113]. *BRCA1* and *BRCA2* work together as tumor suppressors. The ability to properly repair DNA is impaired when either gene is mutated, which increases the risk of genomic instability and, especially, ovarian and breast cancers. To preserve genomic stability and guarantee successful DNA repair, *BRCA1* and *BRCA2* must interact. Together, these genes play a crucial role in maintaining cellular health and protecting from cancer by preventing the accumulation of mutations that can cause the disease.

## 5. Poly (ADP-Ribose) Polymerase (PARP) Molecules

**Figure 1:**
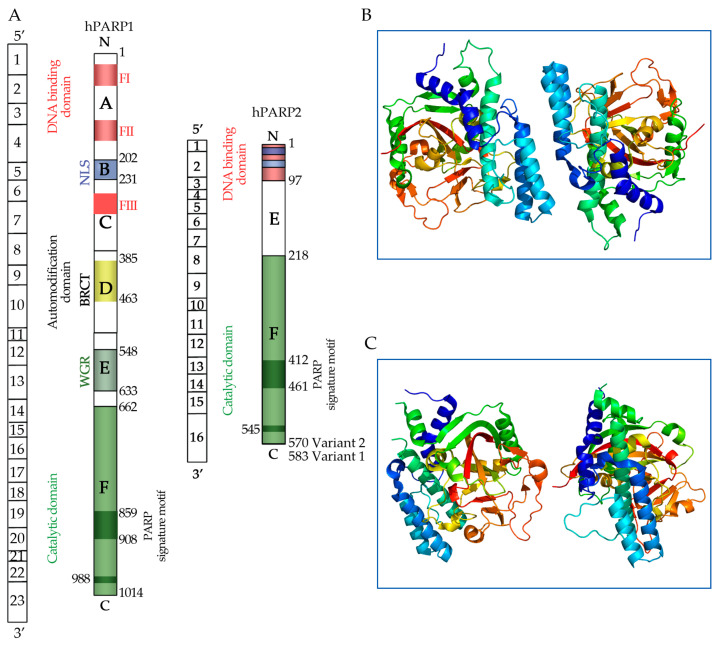
(**A**) A diagrammatic depiction of the protein domains and gene organization of human PARP1 and PARP2. The darkened green box inside the catalytic domain represents the region that is homologous to the PARP signature (residues 859–908 of PARP1 and 412–461 of PARP2 in variant 2) and the essential residue for polymerase activity (glutamic acid 988 of PARP1 and glutamic acid 545 of PARP2 in variant 2). Image modified from Yelamos et al. [115]. (**B**) Poly (ADP-ribose) polymerase 1 (*PARP1*) and (**C**) poly (ADP-ribose) polymerase 2 (*PARP2*) structures. Courtesy of Wikipedia [116,117]. Abbreviations: BRCT: BRCA1 C-terminus motif; WGR: domain with unknown function; FI, FII: zinc fingers motifs; FIII: zinc ribbon domain; NLS: nuclear localization signal; NoLS: nucleolar localization signal. FI and FII red-wine color denotes the zinc finger motifs; the FIII red color indicated the zin ribbon domain; the yellow color represents the BRCA1 carboxy-terminal (BRCT) motif via which PARP-1 participates in protein-protein interactions within the auto-modification domain; the green color denotes the catalytic domain, and the dark green color indicates the PARP signature motif.

The enzyme family known as PARP molecules is involved in some cellular functions associated with DNA repair, cell signaling, and programmed cell death (apoptosis) [114]. PARP is a complex enzyme primarily composed of ADP-ribose units, which are linked to phospholipid groups and ribose sugars, forming a branched polymer (Figure 1).

Its structure includes multiple functional domains that facilitate its role in detecting and repairing DNA damage. The C-terminus is essential for auto-modification and interactions with other proteins, while the N-terminus is critical for DNA binding [118]. PARP consists of two main regions: the NAD^+^ binding site and a protein domain, which includes three unique subdomains: zinc finger, catalytic, and auto modification domains. The zinc finger domain is unnecessary for DNA binding, but is important for the catalytic activity of PARP [119]. The catalytic domain transfers ADP-ribose units from NAD^+^ to target proteins, and the auto-modification domain allows PARP to modify itself by forming poly (ADP-ribose) chains, influencing protein interactions and signaling pathways [120]. The C-terminus can generate large poly (ADP-ribose) chains, often enriched with tryptophan, glycine, and arginine residues, which can modulate enzyme activity and cellular responses to DNA damage. The N-terminal domain aids in the localization of DNA damage sites, ensuring efficient repair processes [114]. The most well-known member of this family, *PARP1*, is essential to the healing of DNA single-strand breaks [121]. DNA damage, especially single-strand breaks, is detected by PARP enzymes, which then start the repair process. By adding poly (ADP-ribose) chains to target proteins, they catalyze the recruitment of extra repair factors to the damage site. In addition to repairing DNA, PARP enzymes are involved in several signaling pathways that control how cells react to damage and stress [122].

This includes modulating protein interactions and influencing the activity of other enzymes. The primary location of PARP molecules is the cell nucleus, where they are essential for DNA repair processes [123]. However, cellular conditions can affect their distribution. Near sites of DNA damage, the majority of PARP proteins, particularly *PARP1,* are in the nucleus. Here, they interact with other proteins involved in the DNA damage response and aid in the repair of single-strand breaks. PARP proteins are predominantly nuclear, but they are also present in the cytoplasm. Additionally, their cytoplasmic activity might be connected to different signaling cascades and reactions to intracellular stress [124]. According to some studies, certain members of the PARP family may localize to mitochondria, where they may play roles in apoptosis and mitochondrial function [125]. PARP activity can trigger cell death pathways in cases of severe and irreversible DNA damage, aiding in the removal of damaged cells. The synthesis of poly (ADP-ribose) by PARP enzymes requires NAD^+^ as a substrate; this process can deplete cellular NAD^+^ stores, which can affect overall cell metabolism and energy balance [126].

PARP inhibitors have emerged as a major field of cancer therapy because of their function in DNA repair, especially for tumors containing *BRCA1* and *BRCA2* mutations.

## 6. Mechanism of Action of PARP Inhibitors

A class of medications known as PARP inhibitors (PARPis) is mainly used to treat breast cancers, especially those with *BRCA1* and *BRCA2* mutations. An enzyme called PARP helps to mend DNA strand breaks when they occur. When DNA damage occurs, PARP recognizes the break and uses the base excision repair (BER) pathway to help repair it. The process of homologous recombination to repair double-strand breaks in DNA depends on the *BRCA1* and *BRCA2* proteins [127].

**Figure 2:**
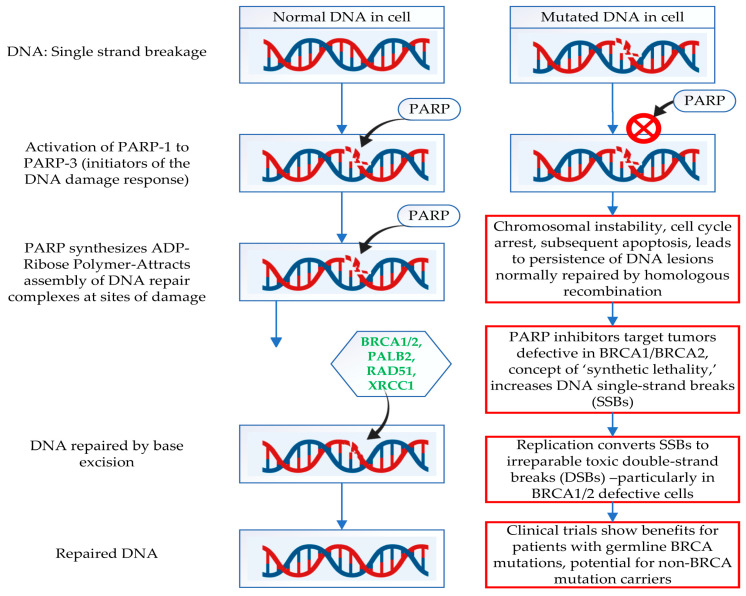
Comparison between normal cell and *BRCA1*/2 mutated cell DNA repair mechanism and the role of PARP molecules. Abbreviations: *BRCA1* = breast cancer gene 1, *BRCA2* = breast cancer gene 2, PALB2 = partner and localizer of *BRCA2*, XRCC1 = X-ray repair cross-complementing protein 1, RAD51 = RAD51 homology1. Red X in circle: Inhibition of PARP.

Figure 2 corresponds to a comparison between normal and *BRCA1*/2 mutated cell DNA repair mechanisms and the role of PARP molecules.

The usual mode of action of PARP inhibitors is the inhibition of poly (ADP-ribose) polymerase (PARP) enzymes, which are important in DNA repair. The novel PARP inhibitors, such as veliparib and rucaparib, can differ in their potency, selectivity, and ability to trap PARP-DNA complexes.

It is possible to compare the modes of action of medications with the authorized PARP inhibitors (olaparib, talazoparib, and niraparib). By inhibiting the *PARP1* and *PARP2* enzymes, olaparib stops DNA single-strand breaks from being repaired. Olaparib prevents the repair of single-strand breaks by inhibiting these enzymes. In cancer cells with defective BRCA1/2 or other defects in homologous recombination repair (HRR), DNA replication results in irreversible double-strand breaks [128].

Other novel PARP inhibitors have been investigated and tried. Several PARP inhibitors that target both *PARP1* and *PARP2* are now being developed, albeit they may also concentrate on blocking other PARP family members, such as *PARP3*. Some improve the effects of PARP trapping to increase the effectiveness of treatment, or to demonstrate specific action targeting *PARP1*. To improve PARP trapping and DNA damage, new inhibitors frequently aim to increase potency or selectivity. To further boost anti-tumor benefits, some are being studied in combination tactics, especially when taken with immune checkpoint inhibitors, chemotherapy, or targeted treatments. Most of the research on these drugs is being conducted in clinical studies that look at different patient groups, combination treatments, and dosage schedules. Their ability to balance potency, toxicity, and selectivity will determine their exact effect.

The ubiquitous post-translational modification of proteins known as poly(ADP-ribosyl)ation, which is catalyzed by PARP, is essential for controlling several cellular functions, including transcription, cell division, proliferation, DNA methylation, and apoptosis [129]. This alteration affects cellular function by modifying the activities of enzymes and the interactions of proteins, DNA, and RNA [130]. For life to continue and for species to remain stable, DNA repair is a fundamental mechanism for maintaining the stability and integrity of the DNA structure [131]. Complex systems for identifying, detecting, and fixing DNA damage are present in cells. Single-strand breaks (SSBs) and double-strand breaks (DSBs) are the two general categories into which DNA damage can be divided [132]. With a heavy reliance on PARP, SSBs are mainly treated by the nucleotide excision repair (NER), base excision repair (BER), and mismatch repair (MMR) pathways [133,134].

## 7. Cell Signaling Pathways of PARP Inhibitors

Inhibitors of PARP mainly target pathways involved in DNA repair, but they also have other effects. An outline of the main cell signaling pathways that PARP inhibitors affect is a sequential event. The Ataxia Telangiectasia Mutated (ATM) and Ataxia Telangiectasia and Rad3-related (ATR) kinases are activated because of increased DNA damage caused by PARP inhibitors [135]. These kinases play a vital role in cellular response regulation and DNA damage recognition. Checkpoint proteins (such as CHK1 and CHK2) become phosphorylated because of ATM/ATR activation, stopping the cell cycle and facilitating repair processes. When there is significant and irreversible damage to DNA, p53 is triggered, which results in cell cycle arrest or apoptosis. In BRCA-deficient cells, PARP inhibitors can increase p53 activation, which triggers apoptotic signaling [136]. Inhibition of PARP can affect cytochrome c release and mitochondrial stress, which can activate caspases and cause apoptosis [137].

**Figure 3:**
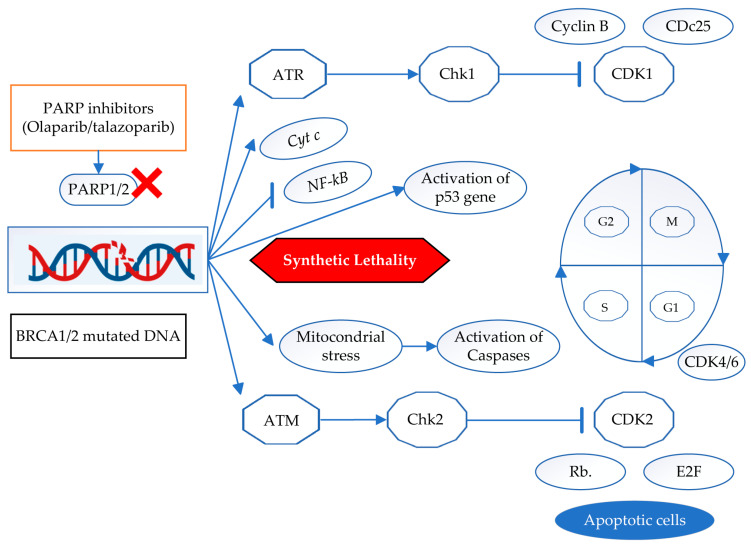
The *BRCA1* and *BRCA2* genes are essential for homologous recombination (HR), which repairs double-strand DNA breaks. However, this repair pathway is compromised and results in genomic instability when *BRCA1* or *BRCA2* is mutated. Single-strand DNA breaks are repaired by the enzymes known as poly (ADP-ribose) polymerase (PARP1/2). Talazoparib and olaparib are examples of PARP inhibitors that block PARP action, preventing single-strand break repair. The cells that have a mutation in *BRCA1*/2 are unable to repair the double-strand breaks caused by these lesions. The buildup of unrepaired DNA damage causes synthetic lethality, which in turn, causes cell death. During this phase, several stress response pathways are activated. Both the Ataxia Telangiectasia Mutated (ATM) and Ataxia Telangiectasia and Rad3-related (ATR) pathways detect DNA damage and initiate the DNA damage response (DDR). Activation of Chk1 and Chk2 inhibits cyclin-dependent kinases (CDK1, CDK2, and CDK4/6), leading to cell cycle arrest. The transcription factors E2F and Rb (retinoblastoma) play a role in facilitating cell cycle arrest and cell death. Apoptosis induction including p53 activation enhances apoptosis (programmed cell death), mitochondrial stress leads to cytochrome c release, which activates caspases, triggering apoptosis, and PARP inhibition disrupts NF-κB-mediated survival signaling, further promoting cell death. Ultimately, these events lead to cell death via apoptosis due to the inability of BRCA1/2-mutated cells to repair accumulated DNA damage. Abbreviations: ATM–Ataxia Telangiectasia Mutated; ATR–Ataxia Telangiectasia and Rad3-related kinases; *BRCA1*/2, breast cancer gene 1/ breast cancer gene 2; Chk1–checkpoint kinase 1; Chk2–checkpoint kinase 2; CDK1–cyclin-dependent kinase 1; CDK2–cyclin-dependent kinase 2; CDK4–cyclin-dependent kinase 4; CDK6–cyclin-dependent kinase 6; Cyt c–cytochrome c; NF-κB–nuclear factor kappa B; Rb–retinoblastoma; E2F–a family of heterodimeric transcription factor. Red X: Inhibition of PARP.

PARP inhibitors may affect the ratio of pro- and anti-apoptotic (BCL-2) and pro-apoptotic (BAX, BAK) proteins, inducing apoptosis in cancer cells [138]. The NF-κB pathway, which is involved in inflammatory responses and cell survival, can be modulated by PARP inhibitors [139]. Because the inhibition interferes with NF-κB-mediated survival signals, it may accelerate cell death. PARP inhibitors cause double-strand breaks in BRCA-deficient cells that are irreversible because there is no functioning HRR. This causes DNA damage to accumulate and eventually results in cell death. In certain situations, PARP inhibitors may also cause cellular senescence, a permanent cell cycle arrest state that can be brought on by significant DNA damage. There is growing evidence that PARP inhibitors can influence autophagy, a process that, depending on the situation, can either induce cell survival or cause cell death. Complex and interconnected signaling pathways are triggered by PARP inhibitors, and in cancer cells, especially those with compromised DNA repair mechanisms, this ultimately results in increased DNA damage, cell cycle arrest, and apoptosis. Knowing these pathways can help develop treatment plans and find biomarkers to help choose patients for PARP inhibitor therapies and other cancers because of their capacity to cause synthetic lethality in BRCA-deficient cells. Figure 3 depicts the *BRCA1*/2-mutated and PARP-inhibited cancer cell signaling mechanisms.

The licensed PARP inhibitors (olaparib, talazoparib, and niraparib) and the ones now being studied (veliparib, rucaparib, and others) have different pharmacokinetics, side-effect profiles, clinical indications, and mechanisms of action. Olaparib primarily inhibits the *PARP1* and *PARP2* enzymes, which stops DNA repair. Single-strand DNA breaks cannot be fixed when these enzymes are suppressed, leading to double-strand breaks and ultimately cell death, particularly in cancer cells that rely on this repair mechanism. Prostate, pancreatic, breast, and ovarian cancers linked to *BRCA1*/2 mutations have all been authorized for it [128]. Olaparib’s half-life is moderate, and it is recommended to take the drug twice a day, although olaparib can cause common side effects such as fatigue, nausea, anemia, and neutropenia [140]. Both *PARP1* and *PARP2* are inhibited by talazoparib. Trapping the PARP-DNA complex increases the potency of the complex and enhances its therapeutic advantages, making it more effective at inhibiting *PARP1* and causing DNA damage [141]. Therefore, it might be proposed that BRCA mutations in breast cancer can be treated with it. Like olaparib, talazoparib can produce fatigue, anemia, and thrombocytopenia; but, due to its stronger strength, side effects could be more severe [142].

Niraparib inhibits both *PARP1* and *PARP2*, but what makes it unique is how long it remains in cells. It can continue to block PARP even after the drug has been taken out of the system because of its long half-life. Regardless of BRCA status, it is approved for ovarian cancer; nevertheless, it is most effective when there are BRCA mutations or homologous recombination deficits (HRDs) [118,143]. Furthermore, it typically needs to be taken once a day and has a long half-life, raising the question of cumulative toxicity from extended use. Additionally, it may cause thrombocytopenia, anemia, and exhaustion [144].

Inhibiting *PARP1* and *PARP2*, rucaparib works similarly to olaparib and niraparib. However, it can trap PARP more efficiently at the DNA site, which, in certain circumstances, may lead to stronger anti-cancer effects [145]. It is used to treat ovarian and prostate cancers with BRCA mutations. Additionally, it is being researched for several cancers, particularly with other therapies. It can be taken twice daily due to its light half-life. Rucaparib, like the other PARP inhibitors, can cause anemia, tiredness, and gastrointestinal problems [146]. It has significant hematologic side effects, but a toxicity profile simpler to manage than talazoparib’s. Olaparib, talazoparib, and niraparib have been approved based on strong evidence from clinical trials in specific cancer types. Their efficacy is especially apparent in cancers with BRCA mutations and limitations in homologous recombination repair [147]. Clinical trials are being conducted to see whether rucaparib can treat various cancers, especially when used with other treatments, since it has shown promise in treating prostate and ovarian cancers [148].

## 8. Clinical Implications

Olaparib, talazoparib, and niraparib are examples of PARP inhibitors that work especially well for patients with hereditary breast and ovarian cancer syndromes caused by BRCA mutations [149]. To increase the effectiveness of therapy, they are frequently used with other medical procedures like immunotherapy and chemotherapy [150]. Patients who may benefit from PARP inhibition can be identified by the presence of BRCA mutations or other deficiencies in DNA repair pathways (e.g., mutations in other homologous recombination-related genes). PARP inhibitors offer a novel therapeutic option for cancers whose DNA repair mechanisms are compromised [151].

They have greatly advanced the treatment of breast cancer. In addition to olaparib, talazoparib, and niraparib, more PARP inhibitors are being researched for the treatment of breast cancer. Rucaparib was first created to treat ovarian cancer, but it is now being investigated for its potential to treat breast cancer, especially in those with BRCA mutations. Although veliparib has been studied mainly with chemotherapy, it is also being considered for the treatment of breast cancer. Research is continuing, and as our knowledge of cancer treatment advances, new compounds might appear. Oncology research on *BRCA1* and *BRCA2* mutations and their association with PARP inhibitors is still very active, especially in cases of breast and ovarian cancer. Recent research has revealed that people with *BRCA1*/2 mutations are not the only ones being investigated for PARP inhibitors like olaparib, niraparib, and rucaparib. The first approved PARP inhibitor, olaparib, is used in the post-chemotherapy phase of advanced breast cancer treatment and shows promising clinical outcomes. Patients with HER2-negative metastatic breast cancer (mBC) that has spread and BRCA mutations benefit most from it [152].

The strong affinity of talazoparib for PARP makes it a more potent PARP inhibitor than olaparib, more effective against BRCA-mutant breast cancer, and longer-acting [153,154]. Niraparib is a potent inhibitor that can be used regardless of a patient’s BRCA status because it may also affect other DNA repair pathways [155]. Similar to olaparib and talazoparib, rucaparib is effective against cancers with BRCA mutations. It has proven effective in treating both ovarian and breast cancers and is used after patients have had previous treatments [156].

A list of PARP inhibitors, along with their therapeutic names and their application fields, is provided in Table 1.

Additionally, they are being studied in tumors with homologous recombination deficiency (HRD), which might benefit from comparable therapeutic modalities. To increase efficacy and overcome resistance, there is growing interest in combining PARP inhibitors with other treatments, such as immunotherapies and targeted agents. Current research focuses on controlling PARP inhibitor side effects and enhancing the quality of life of patients while they are receiving treatment. Trials are being conducted to investigate the efficacy of PARP inhibitors in nascent stages and diverse forms of cancer. A specific area of interest is their application in neoadjuvant environments. The field of genetic testing is changing, and recommendations are being made for more comprehensive testing for HRD in different types of cancer. This could help identify more patients who could benefit from PARP inhibitors. Patients are receiving more and more education about their genetic status, available treatments, and the consequences of having a BRCA mutation.

## 9. Bioinformatic Analysis of Clinical Datasets

### 9.1. Correlation Analysis Between PARP1/2 and Genes Such as ESR1, PGR, ERBB2, BRCA1, and BRCA2 Expression Levels in Breast Cancer Subtypes

**Figure 4:**
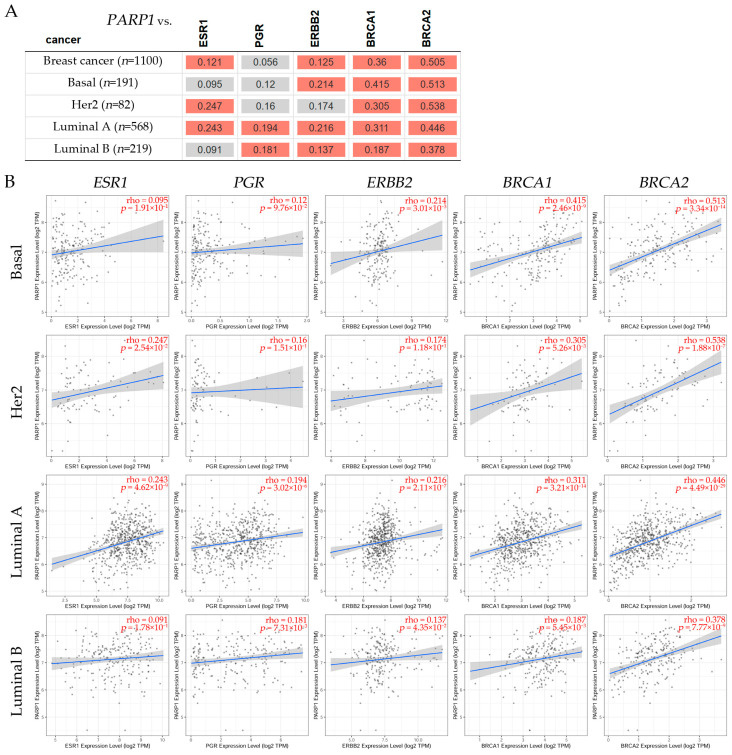
(**A**) The table shows the correlation between *PARP1* and genes such as the estrogen receptor alpha (*ESR1*), the progesterone receptor (*PGR*), the erb-b2 receptor tyrosine kinase 2 (*ERBB2*), the BRCA1 DNA repair associated (*BRCA1*), and the BRCA2 DNA repair associated (*BRCA2*). The red color indicates a significant positive (Spearman’s, *p* < 0.05), and the gray has a non-significant correlation. (**B**) Representative scatter plots show the correlations (Spearman’s, *p* < 0.05) between *PARP1* and *ESR1*, *PGR*, *ERBB2*, *BRCA1*, and *BRCA2* expression levels in breast cancer subtypes such as basal, HER2, luminal A, and luminal B. These plots display linear regression lines and correlation coefficients (ρ) in red in the upper right corner. Each plot shows a blue line indicating the linear regression fit that marks the association between *PARP1* and *ESR1*, *PGR*, *ERBB2*, *BRCA1*, and *BRCA2* expression levels, respectively. The shaded area in grey denotes the confidence interval. Data obtained from TIMER2.0, accessed on 7 November 2024 [157].

**Figure 5:**
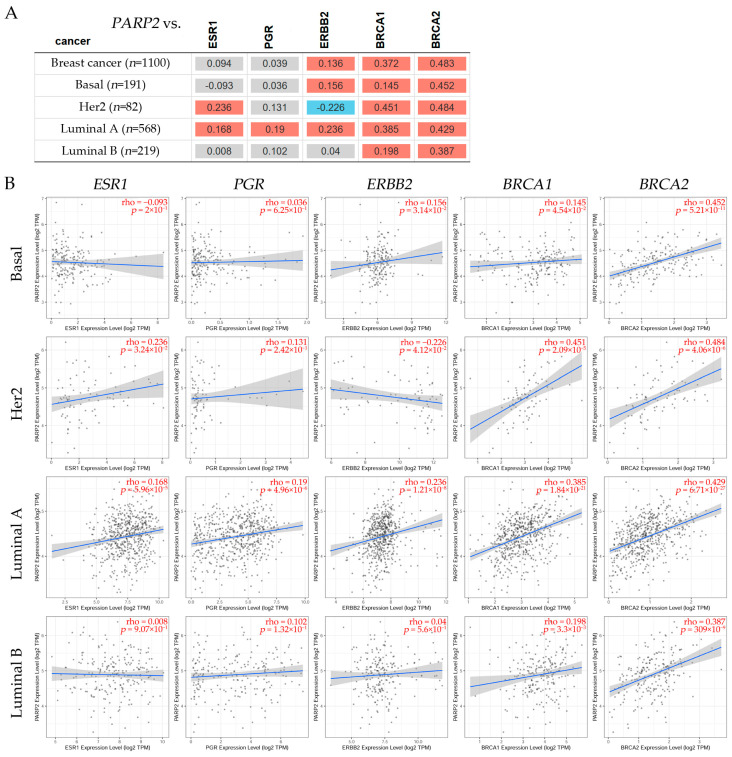
(**A**) The table shows the correlation between *PARP2* and genes such as the estrogen receptor alpha (*ESR1*), the progesterone receptor (*PGR*), the erb-b2 receptor tyrosine kinase 2 (*ERBB2*), the BRCA1 DNA repair associated (*BRCA1*), and the BRCA2 DNA repair associated (*BRCA2*). The red color designates a significant positive correlation (Spearman’s, *p* < 0.05), blue indicated a significant negative correlation (Spearman’s, *p* < 0.05), and gray stands for a non-significant result. (**B**) Representative scatter plots show the correlations (Spearman’s, *p* < 0.05) between PARP2 and ESR1, PGR, ERBB2, BRCA1, and BRCA2 expression levels in breast cancer subtypes such as basal, HER2, luminal A, and luminal B. These plots display linear regression lines and correlation coefficients (ρ) in red in the upper right corner. Each plot shows a blue line indicating the linear regression fit that marks the association between *PARP2* and *ESR1*, *PGR*, *ERBB2*, *BRCA1*, and *BRCA2* expression levels, respectively. The shaded area in grey denotes the confidence interval. Data obtained from TIMER2.0, accessed on 7 November 2024 [157].

To investigate the association between *PARP1*/2 and the estrogen receptor alpha (*ESR1*), *PGR*, *ERBB2*, *BRCA1*, and *BRCA2* genes, a correlation assessment was conducted using the Tumor Immune Estimation Resource v2.0 (TIMER2.0) dataset (Figure 4 and Figure 5). The Gene Correlation module of TIMER2.0 was used to explore such correlations across several breast cancer subtypes, and the statistical analysis was facilitated by Spearman’s test.

It can be observed in Figure 4A a positive correlation between *PARP1* and *BRCA1, BRCA2* in basal, *HER2*, luminal A, and luminal B patients. A positive correlation between *PARP1* and *ERBB2* was found in basal, luminal A, and luminal B, but was non-significant in HER2 patients. On the other hand, there was no significant correlation between *PARP1* and *ESR1* in basal or LumB, but there was a significant correlation between *PARP1* and HER2 and luminal A breast cancer patients. There was also a significant correlation between *PARP1* in *PGR* gene expression in luminal A and luminal B patients. It is concluded from these studies that *PARP1* gene expression is correlated with subtypes in breast cancer patients such as the basal subtype, and it can be positive for *BRCA1*/2 and *ERBB2* gene expression, but is non-significant for *ESR1* and *PGR*, given the opportunity to give specific drugs for such genes; the HER2 subtype can be positive for *BRCA1*/2 and *ESR1*; the luminal A subtype can be positive for all genes mentioned above, including *BRCA1*/2, *ESR1*, *PGR*, and *ERBB2*; and the luminal B subtype can be positive for *BRCA1*/2, *PGR*, and *ERBB2*, but not for *ESR1* gene expression.

In Figure 4B, representative scatter plots show the correlations (Spearman’s, *p* < 0.05) between *PARP1* and *ESR1*, *PGR*, *ERBB2*, *BRCA1*, and *BRCA2* expression levels in breast cancer subtypes such as basal, HER2, luminal A, and luminal B. Results indicated that the basal subtype had non-significance between *PARP1* and *ESR1*, *PGR*, but was positive for *ERBB2* and *BRCA1*/2. The HER2 subtype showed non-significance between *PARP1* and *PGR* and *ERBB2*, but was positive for luminal A and luminal B. The luminal A subtype showed positive significance between *PARP1* and *ESR1*, *PGR*, *ERBB2*, *BRCA1*, and *BRCA2* expression levels, but negative significance in luminal B subtypes. A positive correlation can be seen between *PARP1* and *BRCA1, BRCA2* in basal, *HER2*, luminal A, and luminal B patients. A positive correlation between *PARP1* and *ERBB2* was found in basal, luminal A, and luminal B, but was negative in HER2 patients. However, there was no significant correlation between *PARP1* and *ESR1* in basal or luminal B, but there was a significant correlation in HER2 and luminal A breast cancer patients. There was also a significant correlation between *PARP1* and *PGR* gene expression in luminal A and luminal B patients.

Figure 5A shows the correlation between *PARP2* and genes such as *ESR1*, *PGR*, *ERBB2*, *BRCA1*, and *BRCA2*. The red color indicates a significant positive (Spearman’s, *p* < 0.05), and the gray has a non-significant correlation. It can be observed in Figure 5A a positive correlation between *PARP2* and *BRCA1*, *BRCA2* and with *ERBB2* in the basal subtype; a positive correlation between *PARP2* and *BRCA1*, *BRCA2* and with *ESR1*, but a negative correlation with *ERBB2* in the HER2 subtype; and a positive correlation between *PARP2* and *BRCA1*/2, *ERBB2*, *PGR*, and *ESR1* in the luminal A subtype. However, there was no significant correlation between *PARP2* and *ESR*, *PGR*, and *ERBB2*, being positive with *BRACA1/2* in luminal B breast cancer patients. In Figure 5B, representative scatterplots show the correlations (Spearman’s, *p* < 0.05) between *PARP2* and *ESR1*, *PGR*, *ERBB2*, *BRCA1*, and *BRCA2* expression levels in breast cancer subtypes such as basal, HER2, luminal A, and luminal B. Results indicated that the basal breast cancer subtype had non-significance between *PARP2* and *ESR1*, *PGR*, but was positive for *ERBB2*, *BRCA1*/2. The HER2 subtype showed non-significance between *PARP2* and *PGR*, a negative association with *ERBB2*, and a positive association with *BRCA1*/*2*. The luminal A subtype showed positive significance between *PARP2* and *ESR1*, *PGR*, *ERBB2*, *BRCA1*, and *BRCA2* expression levels, whereas the luminal B subtypes showed a positive association with *BRCA1*/*2*, but was non-significant with *ESR1*, *PRG*, and *ERBB2* breast cancer patients.

### 9.2. Comparative Assessment of PARP1 and PARP2 Expression Levels in Tumor and Normal Tissues in Breast Cancer Subtypes

**Figure 6:**
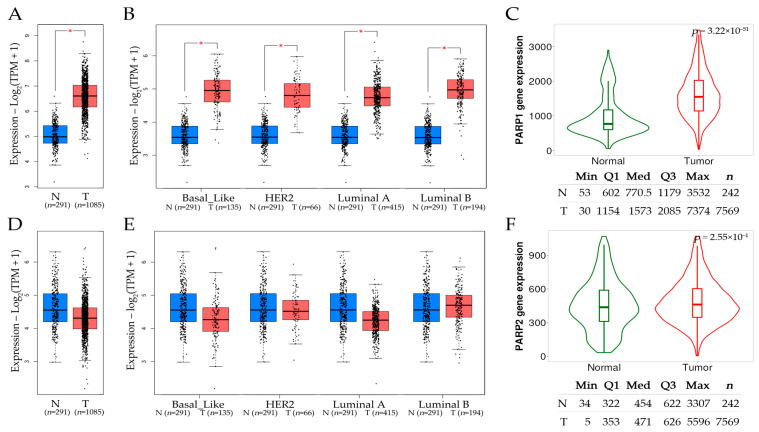
(**A**) Comparison of *PARP1* expression in normal (*n* = 291) and tumor (*n* = 1085) tissues. (**B**) Expression level of *PARP1* in several breast cancer subtypes such as basal-like, HER2, luminal A, and luminal B, including normal tissues. (**C**) Violin plot presenting *PARP1* expression level with normal (*n* = 242) and tumor (*n* = 7569) tissues. (**D**) Comparison of *PARP2* expression in normal (*n* = 291) and tumor (*n* = 1085) tissues. (**E**) Expression level of *PARP2* in several breast cancer subtypes such as basal-like, HER2, luminal A, and luminal B, including normal tissues. (**F**) Violin plot presenting *PARP2* expression level with normal (*n* = 242) and tumor (*n* = 7569) tissues. Data (**A**,**B**,**D**,**E**) obtained from the GEPIA2 dataset [158], accessed on 7 November 2024; violin data (**C**,**F**) retrieved from the TNMplot dataset [159], accessed on 8 November 2024. Abbreviations: N: normal, T: tumor * *p* < 0.05. The red color denotes tumor tissue and blue or green indicates normal tissue. Abbreviations, N: Normal tissue, T: Tumor tissue.

An analysis of *PARP1* and *PARP2* gene expression levels across tumor grades in breast cancer can be seen in Figure 6. The Gene Expression Profiling Interactive Analysis (GEPIA2) dataset provided the comparison of gene expression between tumor and normal tissues, with the distribution of gene expression levels represented through box plots. The Student’s *t*-test was used to compare the expression of *PARP1*/2 between tumor tissues and corresponding normal tissues.

A comparison of *PARP1* and *PARP2* mRNA expression levels between (A) normal and tumor breast tissues, as well as between (B) several breast cancer subtypes such as basal-like, HER2, luminal A, and luminal B, including normal tissues, is provided in Figure 6. GEPIA2 and TNMplot datasets were used for this analysis. A statistically significant *PARP1* overexpression was found in breast tumors in comparison with normal tissue (Figure 6A) and across its several subtypes (Figure 6B). A significant upregulation of *PARP1* mRNA was observed (Figure 6C) in tumors compared to controls (*p* = 3.22 × 10^−51^). The median expression was higher in tumors than in the normal tissues. This overexpression may play a part in the aggressiveness and progression of tumors, as evidenced by its correlation with advanced tumor grades and stages. These findings highlight the diagnostic and prognostic usefulness of *PARP1* in breast cancer by demonstrating that it can be investigated as a trustworthy biomarker with excellent specificity at raised cut-off levels. *PARP2* expression levels were non-significant (Figure 6D–F).

### 9.3. PARP1 and PARP2 as Potential Markers in Chemotherapy-Treated Breast Cancer Patients

**Figure 7:**
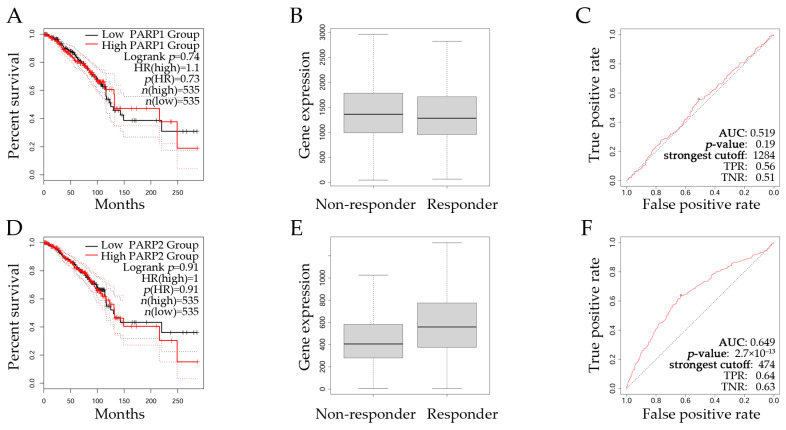
Analysis of *PARP1* and *PARP2* expression levels in breast cancer prognosis and treatment response. Kaplan–Meier (KM) curves presenting the overall survival (OS) of (**A**) *PARP1* expression levels grouping patients into high and low gene expressions. (**B**) Box plots analyzing *PARP1* expression levels between non-responders (*n* = 713) and responders (*n* = 253) to chemotherapy in breast cancer patients. (**C**) Analysis of the ROC curve assessing the predictive power of *PARP1* expression in patients’ chemotherapy responses. Similarly, (**D**) KM curves showing the OS of *PARP2* expression levels grouping patients into high and low gene expressions. (**E**) Box plots analyzing *PARP2* expression levels between non-responders (*n* = 713) and responders (*n* = 253) to chemotherapy in breast cancer patients. (**F**) Analysis of the ROC curve assessing the predictive power of *PARP2* expressions in patients’ chemotherapy responses. Black lines in KM curves indicate low gene expression, and red ones denote high gene expression. KM data (**A**,**D**) obtained from GEPIA2 [158], accessed on 7 November 2024. Responder data (**B**,**C**,**E**,**F**) retrieved from ROC Plotter [160], accessed on 8 November 2024. The black dot in the red line (**C**,**F**) indicates the strongest cutoff, whereas the black dotted line represents no predictive value.

Figure 7 shows an overall survival (OS) analysis of *PARP1* and *PARP2* expression levels in breast cancer patients. GEPIA2 was used to explore the OS of breast cancer patients (Figure 7A,D) by analyzing data from the TCGA, GTEx, and GEO databases; the threshold values for separating the groups with low and high expression were the cut-off low (50%) and cut-off high (50%). The ROC Plotter web platform was used to assess the expression levels of PARP1/2 (Figure 7B,C,E,F, respectively) in breast cancer patients with a pathological complete response (*n* = 1775), treated with chemotherapy and a positive ER status.

Patients were grouped into two categories depending on their PARP expression level: the high expression and low expression groups. OS assessment indicated that patients with high *PARP1* (Figure 7A) and *PARP2* (Figure 7D) expression levels (red line) presented worse survival rates than low PARP expression groups (black lines). Additionally, the receiver operating characteristic (ROC) Plotter dataset was used to assess *PARP1* and *PARP2* expression levels in breast cancer patients whose ER status was positive and who were treated with chemotherapy. Results indicated that responders showed higher mean (598) and median (559) *PARP2* expression levels than non-responders, with a mean value of 454 and median of 406 (Figure 7E). The ROC Plotter showed that *PARP2* had an area under the curve (AUC) of 0.649 presenting a predictive power with a significant *p*-value of 2.7 × 10^−13^ (Figure 7F); the Mann–Whitney test *p*-value (1.8 × 10^−12^) showed a significant difference between both groups, and the fold change was 1.3, implying that responders had increased *PARP2* expression levels. However, *PARP1* was non-significant. Therefore, OS results suggested that *PARP2* expression could be a potential biomarker to predict chemotherapy response in ER-positive patients.

## 10. Conclusions

Since the bioinformatics showed that there was a correlation between PARP1 and BRCA1/2, and at the same time, a correlation with ERBB2 in basal; such results indicate that those breast cancer patients can receive not only PARP inhibitors, but also Anti-HER2 Monoclonal Antibodies as therapeutic drugs. Such analysis by bioinformatics showed that there was a correlation between PARP1 and BRCA1/2, and at the same time, with ESR1 in the HER2 subgroup; those breast cancer patients can receive not only PARP inhibitors, but also tamoxifen or other drugs for positive ESR1 patients as therapeutic drugs. Furthermore, there was a correlation between PARP1 and BRCA1/2, and at the same time, with ERBB2, PGR, and ESR1 in the luminal A subgroup; such results can indicate that those breast cancer patients classified as subgroup luminal A can receive not only PARP inhibitors, but Anti-HER2 Monoclonal Antibodies, tamoxifen, or other drugs for positive ESR1 patients and antiPGR as therapeutic drugs. A correlation was also reported between PARP1 and BRCA1/2, and at the same time, with ERBB2 and PGR in the luminal B subgroup; such results can indicate that those breast cancer patients can receive Anti-HER2 Monoclonal Antibodies and anti-PGR drugs available in the clinic at present.

The bioinformatics study revealed a correlation between PARP2 and BRCA1/2 and ERBB2 in the basal subgroup of breast cancer patients. These patients can receive PARP inhibitors and anti-HER2 Monoclonal Antibodies as therapeutic drugs. There was also a correlation between PARP2 and BRCA1/2 and ESR1 in the HER2 subgroup of breast cancer patients. Those patients can receive not only PARP inhibitors, but also tamoxifen or other therapeutic drugs for positive ESR1 patients. Since bioinformatics showed that there was a correlation between PARP2 and BRCA1/2, ERBB2, PGR, and ESR1 in the luminal A subgroup, patients can receive PARP inhibitors, anti-HER2 Monoclonal Antibodies, tamoxifen, antiPGR as therapeutic drugs, or other drugs for positive ESR1 patients. It was also shown that there was a correlation between PARP2 and BRCA1/2 in the luminal B subgroup, indicating that those breast cancer patients can receive Anti-HER2 Monoclonal Antibodies as therapeutic drugs available in the clinic at present.

On the other hand, through bioinformatics, it was observed that there was a higher overall expression of PARP1 in tumors than in normal tissues and across subtypes, as well as more upregulation of mRNA in tumors than in normal tissues; such results indicate that those breast cancer patients can receive only PARP1 inhibitors. A non-significant difference was found when PARP2 was studied. Concerning survival, results showed that those patients with increased levels of PARP1 and PARP2 expression had worse survival rates than those with low levels. A critical focus is based on finding more selective novel PARP inhibitors that lower toxicity and enhance patients’ overall therapeutic experience. PARP inhibitors frequently work with other treatments, such as immunotherapy and chemotherapy.

Using PARP inhibitors for cancers unrelated to BRCA is a crucial objective. The greatest impact of PARP inhibitors has been demonstrated in BRCA-mutant malignancies, such as ovarian, breast, and prostate cancers, although BRCA mutations only affect a tiny fraction of patients. To increase the number of patients who can benefit from PARP inhibition, clinical investigations are looking into its usage in malignancies with various types of homologous recombination deficit (HRD) and with other treatments. It is yet unknown how to determine which individuals will benefit the most from PARP inhibitors, and not all patients respond to them.

To be constructive in our conclusions, we consider that PARP inhibition has minimal impact on some tumor cells because malignancies are heterogeneous, including cells that may respond differentially to treatment. Research into personalized medicine, where medication is based on individual genetic profiles, would be very beneficial to optimize these combinations and minimize negative effects. Targeted therapy has advanced significantly with PARP inhibitors in the treatment of breast cancer, especially for patients with *BRCA1*/2 mutations. As long as research on combination therapies and treatment protocols is conducted, the future of PARP inhibitors as a mainstay of breast cancer care looks bright. PARPis will play a more significant role in the clinical treatment of cancer. Further research is expected to expand treatment that benefits more patients. Overall, developing PARPis brings new hope to cancer treatment, especially for BRCA mutation-positive patients, as improving the selectivity of PARP-1, enhancing the inhibitory capability of PARPis, and adopting strategies to reduce drug toxicity and side effects are all ideas and directions for developing PARPis.

Future research is needed for the creation of novel PARP inhibitors or other combinations of drugs with enhanced safety and efficacy profiles. Clinical trials and ongoing research will play a critical role in determining this future. Such trials have shown encouraging efficacy, frequently leading to increased response rates and progression-free survival rates in patients with triple-negative breast cancer and other subtypes. There are compelling reasons to keep creating new and enhanced versions. By focusing on distinct pathways or successfully treating malignancies when there is no response to treatments, novel inhibitors may help to overcome this resistance. Today, the most common tumors treated with PARP inhibitors are those associated with BRCA mutations, including breast. More selective novel PARP inhibitors must lower toxicity and enhance patients’ overall therapeutic experience. PARP inhibitors frequently work with other treatments, such as immunotherapy and chemotherapy.

## Figures and Tables

**Table 1:** List of PARP inhibitors, along with their therapeutic names and their application fields.

SL. No	Name of PARP Inhibitors	Stage of Application	References
1	Olaparib	Olaparib, the first PARP inhibitor to be approved, is used in advanced breast cancer treatment after chemotherapy and provides clinically effective results. It is especially useful in patients with BRCA mutations and HER2-negative metastatic breast cancer (mBC).	(Tutt et al., 2021) [152]
2	Talazoparib	Talazoparib is a stronger PARP inhibitor than olaparib, more effective against BRCA-mutant breast cancer, and longer-acting because of its strong affinity for PARP.	(McCann, 2019; Kowalik et al., 2024) [153,154]
3	Niraparib	Developed originally for ovarian cancer, it is being studied for breast cancer. Regardless of a patient’s BRCA status, niraparib is a strong inhibitor that can be used because it may affect other DNA repair pathways.	(Jones et al., 2015) [155]
4	Rucaparib	Like olaparib and talazoparib, rucaparib works well in cancers with BRCA mutations. It is used after patients have undergone prior treatment and has demonstrated efficacy in treating both breast and ovarian cancers.	(Musella et al., 2018) [156]

## Data Availability

TIMER2.0 is freely available at http://timer.cistrome.org/ (accessed on 7 November 2024). GEPIA2, Gene Exploration Profiling Interactive Analysis, is freely available at http://gepia2.cancer-pku.cn/ (accessed on 7 November 2024). TNMplot: differential gene expression analysis in Tumor, Normal, and Metastatic tissues, is freely available at https://tnmplot.com/ (accessed on 7 November 2024). Kaplan-Meier Plotter is freely available at https://www.kmplot.com/ (accessed on 7 November 2024). ROC Plotter is freely available at https://rocplot.org/ (accessed on 8 November 2024). The data generated in the present study may be requested from the corresponding author.
